# Free Accessible Databases as a Source of Information about Food Components and Other Compounds with Anticancer Activity–Brief Review

**DOI:** 10.3390/molecules24040789

**Published:** 2019-02-22

**Authors:** Piotr Minkiewicz, Marta Turło, Anna Iwaniak, Małgorzata Darewicz

**Affiliations:** University of Warmia and Mazury in Olsztyn, Chair of Food Biochemistry, Plac Cieszyński 1, 10-726 Olsztyn-Kortowo, Poland; martaturlo@wp.pl (M.T.); ami@uwm.edu.pl (A.I.); darewicz@uwm.edu.pl (M.D.)

**Keywords:** cancer, neoplasm, natural compounds, food components, databases, chemical information

## Abstract

Diet is considered to be a significant factor in cancer prevention and therapy. Many food components reveal anticancer activity. The increasing number of experiments concerning the anticancer potential of chemical compounds, including food components, is a challenge for data searching. Specialized databases provide an opportunity to overcome this problem. Data concerning the anticancer activity of chemical compounds may be found in general databases of chemical compounds and databases of drugs, including specialized resources concerning anticancer compounds, databases of food components, and databases of individual groups of compounds, such as polyphenols or peptides. This brief review summarizes the state of knowledge of chemical databases containing information concerning natural anticancer compounds (e.g., from food). Additionally, the information about text- and structure-based search options and links between particular internet resources is provided in this paper. Examples of the application of databases in food and nutrition sciences are also presented with special attention to compounds that are interesting from the point of view of dietary cancer prevention. Simple examples of potential database search possibilities are also discussed.

## 1. Introduction

An appropriate diet is considered to be an important factor in cancer therapy and prevention. Therapeutic and preventive role of dietary compounds against cancer is the object of extensive studies, continuously presented in numerous reviews [1,2,3,4]. These reviews include also anticancer applicability of individual substances such as curcumin [5,6], quercetin [7], resveratrol [8,9] or lunasin [10], and individual food resources, such as pomegranate [11,12]. 

The increasing number of experiments concerning data on chemical compounds described in literature is challenging for data retrieval and requires new solutions for this purpose [13,14]. This also concerns anticancer drugs and food components. Bibliographic databases (e.g., Medline, Scopus, Google Scholar) are used as major information resources. An example flowchart of bibliographic database screening was presented by Khan et al. [7].

Specialized databases of chemical compounds, their structures, reactions and biological activity are emerging, but are still under-utilized tools in food science [14,15,16,17,18]. They may be used together with other bioinformatic and cheminformatic tools serving for simulation and prediction of physico-chemical properties as well as biological activity of food components [14,18,19].

Jensen et al. [20,21] have performed high-throughput analysis of the synergistic action of drugs and food components. They provided thousands of examples of the similar activity of drugs and compounds originating from food resources and products. Lacroix et al. [22] analysed the interactions between polyphenols from foods and proteins, including those being drug targets. Naveja et al. [23] supported the above results by finding that several structural and physico-chemical features of drugs and food components are similar. Details concerning particular compounds and their activity and status (activity confirmed by clinical trials, experiment in vivo on animals or in vitro) are summarized, e.g., in chemical and biochemical databases. The databases may thus be helpful tools for food science and education. In our previous publication [16], we presented general information concerning databases of compounds with low molecular weight, their enzymatic reactions and metabolism. The above review also discussed search options, links between databases and examples of their potential application in food and nutrition sciences.

The aim of this review is to provide a brief description of free accessible databases annotating compounds, e.g., food components of interest in the context of anticancer properties.

## 2. Medical, Chemical, Biochemical and Food Databases

The web addresses of selected free accessible chemical, medical and biochemical databases are summarized in Table 1. Data concerning compounds with experimentally proven or potential anticancer activity may be found in databases of drugs (e.g., specialized databases of anticancer drugs). Such databases, among others, annotate natural compounds including those occurring in food resources. Specialized medical databases are focused on biomedical data concerning given substances (biological test results), whereas data concerning physico-chemical properties of particular compounds are available via links to general databases such as PubChem [24] or ChemSpider [25].

The Clinical Trials database includes data concerning synthetic drugs and compounds of natural origin. Curcumin may serve as an example of such a substance [26]. Databases concerning food are focused both on the occurrence of particular compounds in various food resources, such as FooDB, and on human health effects, such as NutriChem [27]. The latter summarizes the information concerning interactions of drugs and food components, including synergistic activity as well as the influence of food components on drug pharmacokinetics (time-dependent changes of drug concentration in the body). PhytoHub annotates the components of plant origin foods. Drug databases also include compounds of food origin. Nutraceuticals belonging to major categories of compounds are annotated in the DrugBank [28].

Databases of individual groups of compounds may also provide information on compounds with anticancer activity. The BIOPEP-UWM (formerly BIOPEP) database of bioactive peptides provides, among others, the largest collection of antioxidative (free radical scavenging) peptide sequences [29]. Free radical scavenging peptides are considered as suitable compounds in cancer prevention, among other health benefits [30]. BIOPEP-UWM also annotates several peptides revealing cytotoxicity against cancer cell lines and other anticancer activities. The above-mentioned database also provides the opportunity to calculate the quantitative parameters describing proteins as potential precursors of bioactive peptides (e.g., possessing anticancer activity) [29,31]. Hydrolysates of food proteins and individual peptides may reveal anticancer activity via various mechanisms [32]. More anticancer peptide sequences together with information concerning biological tests may be found in the CancerPPD database [33]. Polyphenols are considered to be major anticancer and antioxidant food components [34]. They interact with many proteins, including these known as drug targets according to the DrugBank database. Polyphenols are annotated in Phenol-Explorer [35] and databases provided by the US Department of Agriculture (USDA). Some of the carotenoids annotated in the Carotenoids Database [36] also reveal anticancer and/or antioxidant activity.

Databases of enzymes, such as BRENDA [46] provide data about substrates and products of enzymatic reactions, as well as inhibitors and activators of enzymes. Data concerning proteins (enzymes and receptors) as drug targets are also available in both general databases (such as ChEMBL [45]) and specialized drug databases (such as DrugBank [28]). The information about enzymes involved in the metabolism of drugs and other bioactive compounds, including food components, is also available in the KEGG database [38]. Both BRENDA and KEGG are sources of information about the role of particular enzymes in metabolic pathways. Among these two databases, KEGG provides information which is easier to find by the user, whereas BRENDA is more comprehensive. OpenTargets [47] is a specific database containing information about the relationships between enzymes and diseases (e.g., abnormal enzyme activity associated with disease). Annotation of an enzyme in the OpenTargets database together with the term “neoplasm” means, however, only that some experiments concerning this enzyme were performed during research in the area of oncology. Experiments annotated in OpenTargets did not always result in finding any relationships between enzyme activity and cancer. On the other hand, some enzymes in this database are targets for substances with anticancer activity. UniProt [48], the most comprehensive database of protein sequences is applied as a reference database for all databases containing information about enzymes (DrugBank, ChEMBL, KEGG, BRENDA).

Apart from specialized medical databases, some general databases such as PubChem [24] and ChEMBL [45] also contain results of biological tests, including these relevant in the context of neoplasm treatment and prevention (e.g., cytotoxicity against cancer cell lines). The PubChem database also annotates negative test results (tests revealing the lack of expected bioactivity). General databases, especially PubChem, serve as reference databases for more specialized ones (e.g., DrugBank, DrugCentral, KEGG Drug, FooDB). PubChem CID (Compound ID) serves as an unambiguous identifier, recommended for use in research articles and enabling searches using special programs, such as Chemical Translation Service [52].

Metabases such as Labworm or MetaComBio [49] provide access to many databases and other bioinformatic and cheminformatic tools. They are continuously updated and may serve to find, in the future, new databases of interest. Information about categories attributed to particular databases in MetaComBio, presented in Table 1, may facilitate searches in the above metabase. 

Databases presented above offer free access (i.e., without fee) to their content. There are also commercial resources annotating and processing information about chemical compounds (for instance SciFinder, provided by American Chemical Society or OmicTools, provided by omicX company).

## 3. Search Options and Links between Databases

Table 2 shows the search options and links between particular data resources. The advantages and disadvantages of particular search options have been discussed in our previous review [16]. Briefly: there are two general search options: text search (including compound common or chemical names, disease names including particular types of cancer, English or Latin names of plant and animal species being resources of food or natural products, interesting as potential drugs or any terms associated with given compound, resource or disease) and a structure-based search involving annotation of molecules using chemical codes, especially SMILES (Simplified Molecular Input Line Entry Specification) [52] and InChI (International Chemical Identifier) [53] and structures drawn using the molecular editors. The text search is more intuitive but requires knowledge of compound names and other terms in English. The same compound may have several common names. Chemical names of particular compounds, recommended by International Union of Pure and Applied Chemistry (IUPAC) are univocal, but for more complex molecules (e.g., glycosides) they are difficult to be constructed even using specialized programs. The same compound, if it is especially well-known, may have many common or medical names. It may provide a problem with using common names as a query in database searches. The application of InChIKeys [54] as a query may be also considered as a text search. InChIKeys are unambiguous signatures always containing 27 characters. Some of the compounds annotated in the PubChem database [24] are described using InChIKeys instead of common names.

Searching based on compound structure is an emerging strategy [16]. As mentioned previously [16], there are two possible ways for a structure input: drawing via molecule editor or input of a structure written using chemical code (especially SMILES). Some databases (e.g., DrugBank, ChEMBL, ChemSpider, FooDB and PhytoHub) offer a combined opportunity–import of SMILES code via molecule editor and display compound structure. Some molecule editors are able to display inappropriate valence of particular atoms or missed configuration of substituents around asymmetric carbon atoms–typical errors occurring in publicly available molecule structures [55]. An efficient search requires knowledge of compound structures, including stereoisomers. 

Search engines of general, drug and food databases enable finding set of compounds with structures similar to the query molecule. The coefficient proposed by Rogers and Tanimoto [56], entitled the Tanimoto coefficient, seems to be the most popular measure to date for the similarity between two molecules [16,18]. It is usually expected that compounds with a similar molecular structure possess similar biological activity. On the other hand, the possibility of the occurrence of so-called “activity cliffs”, understood as slight changes in structure leading to significant changes of biological activity, should also be taken into account [18,56,57].

The screening of databases of low molecular compounds may be supported by computer applications such as Chemical Translation Service [51]. This program provides the opportunity to convert a chemical name or InChIKey of a compound into ID numbers in many databases (e.g., PubChem and ChemSpider). Translation between SMILES, InChI, InChIKeys and chemical names is possible using the Chemical Identifier Resolver [50] or ChemRTP. The second program is designed for prediction of physico-chemical properties of compounds. Full access to ChemRTP program and associated MolInstints database requires subscription, but option of conversion of SMILES or drawn structure into chemical name, InChI or InChIKey is free-accessible.

Amino acid sequences are the first option of a structure-based search in databases of peptides, proteins and enzymes (e.g., BIOPEP-UWM [29], ChEMBL [45], UniProt [48]). BIOPEP-UWM provides two sequence-based search option: substructure (subsequence) search as the option finding all peptide sequences containing a given fragment as default and exact match as an alternative opportunity, chosen by the user. Databases annotating enzymes, such as BRENDA [46], ChEMBL [45] or UniProt [48] offer the possibility of finding sequences similar to the query sequence. BLAST [58] is the most popular algorithm serving this purpose.

Links between the databases mentioned in this article are presented in Table 2. The significance of such links was emphasized in our previous review [16]. They may be applied for finding information via “navigating the network of links and cross-links between databases” [16]. This option includes the opportunity for the access of a single compound, enzyme or disease data in multiple databases using a single search engine. ChemSpider [25] provides multiple links to data concerning extensively studied and well-known compounds. ChEMBL [45] is another database offering external links. The utility of such links may be illustrated by the following example. The BRENDA database [46] does not provide (December 2018) a search option to find ligand data using SMILES–the most popular chemical code. This gap may be filled by using the ChEMBL search engine and then finding a link to BRENDA. As emphasized in our previous article, the PubChem database is used as a reference data source for other databases, annotating both information concerning drugs and food components. Recently, PubChem has started to utilize external links to data of particular compounds in other resources (e.g., ChemSpider or ChEMBL).

## 4. Examples of Current Applications of Internet Databases in Food and Nutrition Science 

Extensive studies, including mining of data concerning the biological activity of food components of plant origin, have been performed by Jensen et al. [20]. The authors used, among others, ChEMBL [45] and KEGG [38] databases to find bioactive compounds and the metabolic pathways associated with them. A structure-based search utilizing SMILES strings was applied for the ChEMBL screening. The authors found over 20,000 compounds originating from c.a. 16,000 plant species, which may be beneficial in the treatment of c.a. 1600 diseases. Many foods may be considered as promising in the treatment of various kinds of cancer although further experimental studies are necessary to confirm such predictions. A case study on colon cancer has been discussed as an example illustrating the potential of data mining supported by chemical databases. The authors have found 623 compounds from 519 plants, revealing the effect on metabolic pathways, which may potentially lead to suppression of colon cancer. Some of these plants were objects of experimental studies concerning supporting colon cancer therapy by diet. Next 1415 compounds revealed high similarity (using Tanimoto coefficient as similarity measure) to drugs and phytochemicals active against colon cancer. This result may indicate the direction for further experimental work aimed at finding new anticancer compounds, although negative results are also possible due to the presence of activity cliffs. Jensen et al. [20] have pointed out that most of the active compounds occur in many plant species even if there is no close taxonomic relation between them. This finding is consistent with the data summarized in the FooDB database.

Another publication of Jensen et al. [21] concerns the interactions between 1800 drugs and 4000 foods of plant origin. Food components may affect the fate of drugs (including anticancer ones) in the human body. Drug absorption involves, for example, binding with proteins acting as carriers. Drugs may be substrates for enzymes, which convert them into inactive products. Foods may also enhance the shelf life of drugs by inhibition of enzymes catalysing their reactions, for example. The negative effect of foods on drug activity may occur if drug and food components interact with the same carrier. In that case, food components may compete with drugs and thus inhibit their absorption. Anticancer compounds belong to the main classes of drugs with an activity affected by diet. The authors used data from DrugBank [28] and ChEMBL [45] databases. Their results are summarized in the NutriChem [27] database. The above findings served as a basis for designing of dietary recommendations based on knowledge concerning biological activity of food components [59].

A typical example of the application of chemical databases is their use for calculating the intake of compounds of interest. The daily intake of polyphenols in various countries was calculated on the basis of data summarized in the Phenol-Explorer and USDA [60,61,62]. The intake of particular foods was reported by participants. The daily intake of polyphenols was calculated using data concerning diet, received from participants and the polyphenol content in individual food items, annotated in the above databases. 

Phenol-Explorer was also used for the survey of interactions between polyphenols of food origin and proteins [22]. Drug targets, annotated in the DrugBank [28] database, include proteins which are polyphenol interactors. Two protein classes: nuclear receptors and cyclin-dependent kinases, are considered important in the context of cancer development and thus as potential targets of anticancer drugs. On the other hand, it should be noted that interactions of food components with proteins which are drug targets are not always beneficial. As pointed out by Jensen et al. [21], they may also be antagonists of drugs. Naveja et al. [23] have recommended FooDB as an alternative resource for studies involving the structure, properties and content of polyphenols in particular foods.

Terlikowska et al. [26] have summarized the results of clinical trials concerning the anticancer activity of a well-known food component, curcumin and its analogues, on the basis of data retrieved from the ClinicalTrials database. Curcumin and its analogues and derivatives (e.g., dimers and glycosides) are annotated in the FooDB database. Clinical trials have confirmed the anticancer activity of curcumin against primary epithelial ovarian cancer. Moreover, curcumin revealed a synergistic effect with some chemotherapeutics. This compound may also be considered as safe (not revealing significant negative side effects).

An example application of a specialized peptide database–BIOPEP-UWM was described by Borawska et al. [63,64]. The database application included determination of the location of antioxidative fragments within carp (*Cyprinus carpio*) [63] and salmon (*Salmo salar*) [64] protein sequences (building of profiles of potential biological activity of protein fragments [29]) and simulation of proteolysis by human digestive enzymes. All operations were performed using amino acid sequences, annotated using a standard one-letter code. Protein sequences of both fish species were taken from the UniProt database. The above in silico predictions were followed by experimental measurements of antioxidant activity using typical tests utilizing DPPH (1,1-diphenyl-2-picrylhydrazyl), ABTS (2,2-azinobis(3-ethyl)-benzothiazoline-6-sulfonic acid) and ferric reducing power. Peptides predicted to be released by digestive enzymes, as well as these occurring in protein sequences, were identified using reversed-phase high performance liquid chromatography coupled with tandem mass spectrometry (RP-HPLC-MS/MS). Peptide detection was supported by the prediction of MS/MS spectra and retention times. Another possibility is the identification of peptides followed by a database search to identify which ones are bioactive (e.g., antioxidative or cytotoxic).

## 5. Possible Simple Search Schemes to Use for Finding Information Concerning Anticancer Compounds from Food Science 

**Figure 1:**
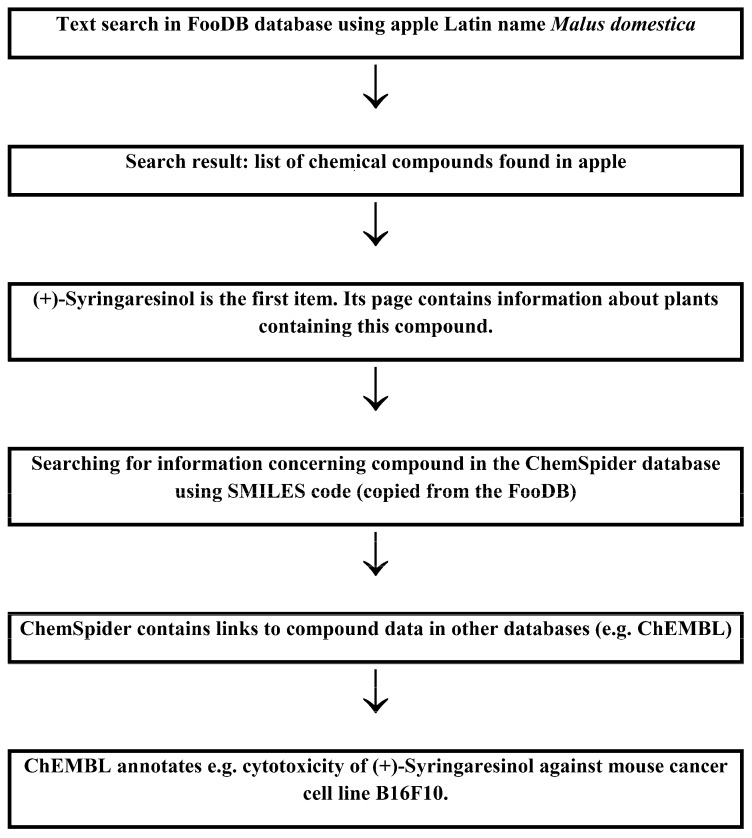
Possible search scheme aimed at finding the anticancer activity of compounds derived from apples (*Malus domestica*) using (+)-Syringaresinol as an example.

**Figure 2:**
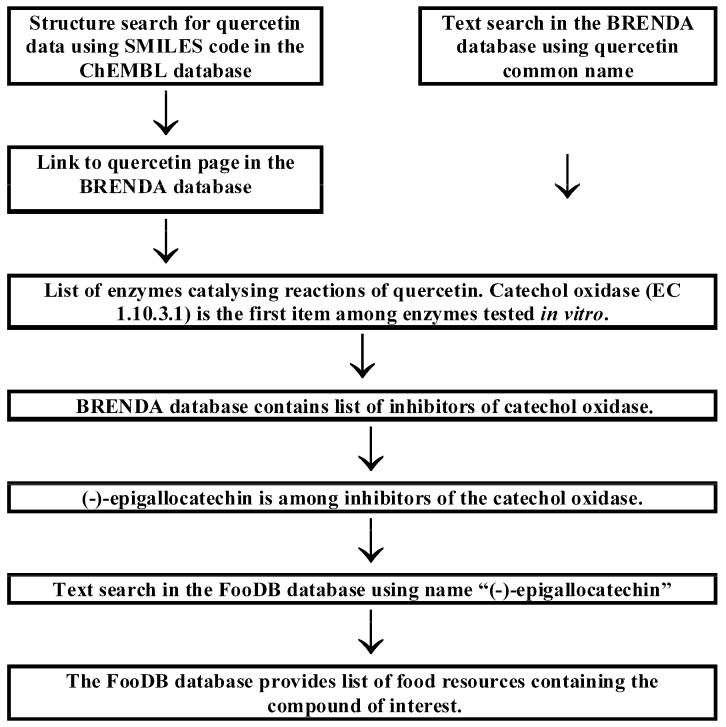
Possible search scheme aimed at finding inhibitors of enzymes catalysing reactions of quercetin.

Two possible search schemes concerning the anticancer activity of food components and the influence of food-derived compounds on the metabolic fate of known anticancer compounds are presented in Figure 1 and Figure 2, respectively.

Figure 1 presents a scheme for searching for the anticancer properties of components of apples. The Latin name *Malus domestica* is used as a query for interrogation of the FooDB database. Apple (Malus pumila) is among the results. There are 314 compounds from apples annotated in the FooDB database. (+)-Syringaresinol (IUPAC chemical name: 4-[(1S,3aR,4S,6aR)-4-(4-hydroxy-3,5-dimethoxyphenyl)-hexahydrofuro[3,4-c]furan-1-yl]-2,6-dimethoxyphenol) belonging to the compound class named lignans, is first of them. Its status in apples is annotated as “detected, not quantified”. It has been found in 18 plants, including barley, common buckwheat, common wheat, garden tomato, grape wine, kiwi, pineapple, rye, sesame and tofu. It was quantified in most of these resources. Compound information in the FooDB database contains direct links to corresponding pages in PubChem [24] and KEGG [38]. A structure-based search using the ChemSpider [25] search engine and SMILES representation as a query provides access to other databases, e.g., ChEMBL. There is an alternative opportunity. SMILES code may be converted into InChIKey using the Chemical Identifier Resolver program. The resulting InChIKey may be used for a Google^TM^ search with similar results. ChEMBL annotates cytotoxic activity against mouse cancer cell lines, as mentioned in Figure 1 and against several lines of human cancer cells in vitro. The ChEMBL compound data card contains a link to (+)-Syringaresinol data in the BRENDA [46] database, providing information on enzymes catalysing its reactions, for example. Such data may be helpful in the prediction of compound metabolism.

Another possible search scheme is presented in Figure 2. It concerns the search for compounds affecting the metabolic fate of anticancer substances using quercetin as an example. The BRENDA [46] database provides comprehensive information on enzymes catalysing reactions of this compound. Users may perform searches directly in the BRENDA database using the text search option with “quercetin” as a query. A structure-based search is possible via the ChEMBL [45] database search engine using SMILES representation. The compound report card in the ChEMBL database offers external links to compound data in several other databases, e.g., BRENDA. This database contains information on the status of quercetin as an enzyme ligand: substrate, product inhibitor or activator. The metabolism of quercetin involves reactions in which it acts as a substrate. Human enzymes are also considered. Catechol oxidase (EC 1.10.3.1) catalyses quercetin oxidation with unknown products. The enzyme was investigated in vitro. BRENDA provides a list of species producing this enzyme, including *Homo sapiens*. Inhibitors of enzymes catalysing quercetin reactions may be expected as factors extending the shelf-life of this compound and thus enhancing its biological activity. (−)-Epigallocatechin is the first compound annotated in BRENDA as an inhibitor of catechol oxidase. The FooDB database provides a list of 26 food resources containing the above compound, e.g., broad bean, pecan nut and tea. The above result of database searching can be considered the starting point for further investigations. Catechol oxidase is annotated as an enzyme catalysing quercetin oxidation in vitro, but its effect in vivo was not reported. The same remark concerns the role of (−)-epigallocatechin as an enzyme inhibitor. It is possible to utilize many search schemes using the databases presented in this review. Moreover, the number of databases available is increasing rapidly.

The use of databases has drawn some criticism. Recommendations concerning the curation of datasets and search procedures include taking into account the strengths and weaknesses of databases and the recognition and correction of possible errors in query structures [54,65,66]. Published opinions about databases may also not be up to date. For example, in our review from 2016 [16] we published the opinion that ChemSpider possesses a simpler search engine than PubChem. The interface of the second database has been significantly modified and is now more user-friendly than in 2016.

Education in the areas of food science, human nutrition and dietetics can utilize the benefits and meet the challenges of the big data era. The potential of Internet databases in teaching chemistry has been recently emphasized by Tuvi-Arad and Blonder [67]. Databases may serve as tools supporting classic, printed handbooks. Molecule structures annotated in databases and datasets are considered to be a specific language. Particular molecule structures (words used as keywords) may be drawn using special programs called “molecule editors” or annotated using computer-readable codes (e.g., SMILES). The use of modern chemistry language to ask questions on the Internet is a useful skill for students of food technology and human nutrition, as a field of study involving chemistry. Data mining from databases classified as chemical, using a structure-based search (i.e., asking questions using chemical language) has been added to courses for food science students at our faculty [16,49].

## 6. Recommendations for Database Choice

Choice of particular database should be done taking into account two crucial factors: database content and search options. Advantages and weak points of some databases relevant for food science were discussed in our previous review [16]. Recommendations for database choice are discussed here using general and medical databases as examples.

Questions concerning database content are as follows. How many compounds are annotated in the database? How comprehensive is information about each compound? Does database contain unique information? How often is the database updated? Does database contain errors in compound structures or in other information?

PubChem database is the largest database of low-molecular compounds. On the other hand, most of compounds annotated in this database are not well-known. ChEMBL contains less compounds than PubChem but provides more information about biological activity of any of them. DrugBank provides most extensive description of drugs among medical and pharmaceutical databases. Information in this database is more extensive than in smaller and more specialized databases of anticancer drugs and compounds. It offers also many external links (see Table 2). On the other hand, brief information may be easier to find and process by the user. For instance, KEGG database provides rapid access to information concerning enzymes and metabolic pathways involving reactions catalyzed by them, whereas BRENDA database offers much more details concerning these enzymes. KEGG may be thus recommended for preliminary search, BRENDA—as a source of more detailed information. ClinicalTrials database is an example of database providing unique information—a systematic review of clinical investigations concerning particular compounds, including both drugs and nutraceuticals. Open Targets provides the most extensive review on association between enzymes (abnormal activity of enzymes) and diseases. The largest databases mentioned in this review (PubChem, UniProt, ChEMBL, DrugBank) are also systematically updated. UniProt includes up to c.a. half million new protein sequences per month. The recent version of DrugBank is the fifth in its history. Various errors (e.g., errors in structure, inappropriate reference citations) occur in all existing databases. They always provide processed “second hand” information. PubChem curators have recently performed standardization of molecule structures [68], intended to help in elimination of errors during submission of compound data. Taking in mind above-mentioned remarks, especially these concerning data uniqueness and occurrence of errors we can recommend confrontation of more than one database to obtain complete and reliable information.

The search engine is the second important factor affecting database usefulness. A potential user should consider three main questions when using specific database. How many search options offers particular database? Is the search engine user-friendly? Is the search engine unfailing i.e., do all available options work properly? If database content justifies acceptance of some problems with search engine there is fourth question: how to overcome or omit these problems?

Text search seems to be more intuitive search option. DrugBank, DrugCentral and NPCARE databases offer search using drug name, protein target name and disease name (in that case cancer type). In the DrugBank search engine disease name search option is labeled as “Indication”. Particular drug databases offer some additional text search options such as pathway (DrugBank) compound class (NPACT), names of genes asociated with cancer (NPCARE) or tissues and cell lines (PharmacoDB). Smaller databases enable relatively rapid browse list of compounds of interest as compared with the larger ones.

Databases mentioned in this review can be divided into two major categories according to Table 2. The first category covers databases providing only text-based search whereas the second one includes both text- and structure-based search. Amino acid sequences are considered as structures for the purpose of this classification. As mentioned previously [16] we recommend the second category. Using the program named molecule editor (molecular editor) is one of the most popular options of query input [16]. Molecular editors allow drawing the molecule structure and convert it into SMILES code. JSME editor [69], used e.g., at the website of Chemical Structure Lookup Service is easy to use and may be recommended for beginners (e.g., for students during classes concerning chemical information [49]). On the other hand, Marvin JS (Provider: ChemAxon, Budapest, Hungary) possesses more options, e.g., displaying of absolute configuration (Rectus or Sinister) around asymmetric carbon atoms (important in the context of work with chiral molecules) and detection of errors such as missed chirality centers (displayed by using “?” character instead of “R” or “S”) or inappropriate valence of particular atoms. The last option makes Marvin JS a useful tool if structure validation and correction of errors is necessary [54]. Moreover, the second of above-mentioned editors offers output of structure in additional formats (e.g., InChI and InChIKey). The Marvin JS Molecule editor is applied in search engines of ChEMBL, DrugBank and FooDB databases.

The search engine of PubChem database is recently an excellent example of simplicity. Search engine is available via the tab "Try the PubChem Search Beta" at the homepage. This search engine offers one window integrating search by text and structure (annotated using SMILES or InChI Code). Search engine accepts compound name, chemical formula, CAS Registry Number, InChIKey (not annotated at list of available options below window serving for query input), SMILES or InChI. Separate icon entitled “Draw structure” enables the access to molecule editor. As mentioned above, we published [16] opinion that search engine of PubChem is complex and less user-friendly as compared with ChemSpider. Recently, simplicity of both search engines is comparable. The "Try the PubChem Search Beta" tab at PubChem website corresponds to the “Simple search” option of ChemSpider whereas PubChem’s “Draw structure”–to “Structure search” option of ChemSpider.

There is one more reason to consider PubChem search engine as excellent. Search options in PubChem and ChemSpider are almost the same, but the first database is more unfailing than the second one. This difference may be illustrated by the following example. There are two versions of aromatic rings annotation in SMILES: Kekulé version (for instance l-tryptophan is annotated as C1=CC2=C(C=C1)[N](C=C2C[C@@H](C(=O)O)N)[H]) and aromatic version (l-tryptophan representation is c1ccc2c(c1)c(c[nH]2)C[C@@H](C(=O)O)N). l-Tryptophan is an example of a molecule containing fused aromatic rings. PubChem search engine accepts both kinds of SMILES representations of compounds containing fused aromatic rings (e.g., tryptophan and peptides containing this amino acid, polyphenols etc.). ChemSpider search engine accepts only Kekulé version. This problem may be omitted using InChI or InChIKey as a query for the “Simple search” option. Use of InChI representation of L-tryptophan as a query for structure search results (14 February 2019) in finding tryptophan with missed chirality center (without defined configuration around asymmetric carbon atom–DL-tryptophan). Any search option in PubChem using InChI or aromatic version of SMILES leads to appropriate result i.e., finding l-tryptophan data. Moreover, PubChem molecule editor automatically generates Kekulé version of SMILES representation of aromatic molecule on the basis of InChI or aromatic SMILES representation. Kekulé version of SMILES string of molecule with fused aromatic rings may be successfully used for search in ChemSpider. This advantage of PubChem is a result of recently performed standardization of aromatic molecule representations based on Kekulé version of SMILES [68].

If the search engine does not offer some opportunity (e.g., structure-based search), the user may look for the possibility of using another search engine following by link to database of interest. For instance, data annotated in ClinicalTrials and BRENDA databases may be available via the ChEMBL search engine using both text and structure-based search. Links to compound data in KEGG are available at ChemSpider, DrugBank and FooDB websites. ChemSpider, ChEMBL, DrugBank and FooDB offer access to data in many other information sources.

## 7. Final Remarks

Databases can enhance access to data concerning chemical compounds with biological activity, making them interesting from the point of view of designing diets supporting anticancer therapy. This advantage is important in the “Big Data Era”. On the other hand, information from databases cannot replace experiments in the creation of new knowledge, although information retrieved may identify gaps in the existing information and show the direction for the design of new research.

Chemical compound databases are commonly used in chemical, pharmaceutical, and biological sciences. Their use in food and nutrition sciences is not as extensive as in the above-mentioned areas. Examples presented here suggest that databases may be helpful tools in both research and education. Role of the databases is expected to increase in the near future.

## Figures and Tables

**Table 1:** Web addresses of databases containing information about anticancer compounds.

Database	Website	Reference	Provider
General databases of drugs (MetaComBio category: “Pharmacologically active compounds”)			
ClinicalTrials	https://clinicaltrials.gov/	*	National Institutes of Health, Bethesda, MD, USA
DrugBank	https://www.drugbank.ca/	[28]	University of Alberta, Edmonton, Canada
DrugCentral	http://drugcentral.org/	[37]	University of New Mexico, Albuquerque, NM, USA
KEGG drug	http://www.genome.jp/kegg/drug/	[38]	Kyoto University, Kyoto, Japan
SuperDrug	http://cheminfo.charite.de/superdrug2/	[39]	Charité University of Medicine, Berlin, Germany
Specialized databases of anticancer drugs, including natural products (MetaComBio category: “Pharmacologically active compounds”)			
CancerResource	http://data-analysis.charite.de/care/	[40]	Charité University of Medicine, Berlin, Germany
canSAR	http://cansar.icr.ac.uk/	[41]	Institute of Cancer Research, London, UK
NPACT	http://crdd.osdd.net/raghava/npact/	[42]	Institute of Microbial Technology, Chandigargh, India
NPCARE	http://silver.sejong.ac.kr/npcare/	[43]	Sejong University, Seoul, South Korea
PharmacoDB	https://pharmacodb.pmgenomics.ca/	[44]	Princess Margaret Cancer Centre, Toronto, Canada
General databases of food components (MetaComBio category: “Food components”)			
FooDB	http://foodb.ca/	*	University of Alberta, Edmonton, Canada
NutriChem	http://147.8.185.62/services/NutriChem-2.0/	[27]	The University of HongKong, Hong Kong
PhytoHub	http://phytohub.eu/	*	Institut National de la Recherche Agronomique, Paris, France
USDA Food Composition Databases	https://ndb.nal.usda.gov/ndb/search/list	*	US Department of Agriculture, Washington, DC, USA
Databases of individual classes of compounds (e.g., from food), revealing antioxidative and anticancer activity (MetaComBio categories: “Food components”, “Amino acids and peptides”, “Lipids”, “Phenolic compounds”)			
BIOPEP-UWM	http://www.uwm.edu.pl/biochemia/index.php/pl/biopep	[29,31]	University of Warmia and Mazury in Olsztyn, Olsztyn, Poland
CancerPPD	http://crdd.osdd.net/raghava/cancerppd/index.php	[33]	Institute of Microbial Technology, Chandigargh, India
Carotenoids Database	http://carotenoiddb.jp/	[36]	National Institute of Genetics, Mishima, Japan
Phenol-Explorer	http://phenol-explorer.eu/	[35]	Institut National de la Recherche Agronomique, Lyon, France
USDA Flavonoids	https://data.nal.usda.gov/dataset/usda-databaseflavonoid-content-selected-foods-release-32-november-2015	*	US Department of Agriculture, Washington, DC, USA
USDA Isoflavones	https://data.nal.usda.gov/dataset/usda-database-isoflavones-isoflavone-content-selected-foods-release-21-november-2015	*	US Department of Agriculture, Washington, DC, USA
USDA Proanthocyanidins	https://data.nal.usda.gov/dataset/usda-databaseproanthocyanidin-content-selected-foods-release-2-2015	*	US Department of Agriculture, Washington, DC, USA
General databases of compounds with low molecular weight (MetaComBio category: “Miscellaneous compounds”)			
ChEMBL	https://www.ebi.ac.uk/chembldb/	[45]	European Bioinformatics Institute, Hinxton, UK
ChemSpider	http://www.chemspider.com/Default.aspx	[25]	Royal Society of Chemistry, London, UK
PubChem	https://pubchem.ncbi.nlm.nih.gov/	[24]	National Center for Biotechnology Information, Bethesda, MD, USA
Databases of enzymes (MetaComBio category: “Biochemical reactions”)			
BRENDA	http://www.brenda-enzymes.org/	[46]	Technical University of Braunschweig, Braunschweig, Germany
OpenTargets	https://www.targetvalidation.org/	[47]	International group “OpenTargets Consortium”, Hinxton, UK
Database of proteins			
UniProt	http://www.uniprot.org/	[48]	European Bioinformatics Institute, Hinxton, UK
Metabases (MetaComBio category: “Metabases”)			
LabWorm	https://labworm.com/	*	Independent group, Jerusalem, Israel
MetaComBio	http://www.uwm.edu.pl/metachemibio/index.php/about-metacombio	[49]	University of Warmia and Mazury in Olsztyn, Olsztyn, Poland
Programs supporting database search (MetaComBio category: “Programs”)			
Chemical Identifier Resolver	https://cactus.nci.nih.gov/chemical/structure	[50]	National Cancer Institute; Bethesda, MD, USA
Chemical Translation Service	http://cts.fiehnlab.ucdavis.edu/	[51]	University of California Davis, Davis, CA, USA
ChemRTP (MolInstincts) **	http://www.chemrtp.com/	*	ChemEssen Inc. Seoul, South Korea

* No reference available. ** Commercial resource offering free access to part of its content. All above resources were accessed between October 2018 and February 2019.

**Table 2:** Search options and links between databases containing information about anticancer compounds.

Database	Search Options	Links *
General databases of drugs		
ClinicalTrials	Text search: drug names	Links from ChEMBL
DrugBank	Text search: drug names, target names, metabolic pathways, disease names (indications). Search may include filters, e.g., “nutraceuticals” Structure search: input using SMILES or InChI codes; input via molecular editor, opportunities for exact, similarity or substructure search	Links from ChemSpider, KEGG, SuperDrug, FooDB Links to many databases, e.g., PubChem, KEGG, ChEMBL, ChemSpider, UniProt **
DrugCentral	Text search: drug names; target names, disease, pharmacologic action	Links from SuperDrug Links to ChEMBL, UniProt **, DrugBank, KEGG Drug, PubChem
KEGG drug	Text search: drug names	Links from ChemSpider, DrugCentral; PubChem; SuperDrug Links to PubChem, BRENDA
SuperDrug	Text search: drug names Structure search: structure input via molecular editor, possible import of mol (mdl molfile) format to the editor; opportunities for exact match or search by similarity	Links to UniProt **, DrugBank, DrugCentral, KEGG Drug, ChEMBL and PubChem
Specialized databases of anticancer drugs		
CancerResource	Text search: drug name; PubChem CID; target name, target protein no. in UniProt Structure search: SMILES code or structure drawn using molecular editor, search by similarity	Links to PubChem and UniProt **
canSAR	Text search: drug name, target name	Links to ChEMBL and UniProt **
NPACT	Text search: compound name, compound class, PubChem CID; InChIKey Structure search: SMILES code	Links to PubChem and UniProt **
NPCARE	Text search: cancer type, gene name of target protein, genus organism being source of compound	Links to PubChem and UniProt **
PharmacoDB	Browse list of compounds, tissues, cell lines and targets Text search: drug names	No links
General databases of food components		
FooDB	Text search: names of compounds, food products and organisms; Structure search: SMILES or InChI, molecular editor, similarity search	Links from ChemSpider; Links to ChemSpider, PubChem, DrugBank, ChEMBL, Phenol Explorer, KEGG
NutriChem	Text search: compound and disease name Structure search: SMILES, InChI or molecular editor	No links
PhytoHub	Text search: compound name, food name, molecular formula Structure search: SMILES, InChI or molecular editor	Links to PubChem
USDA Food Composition Databases	Text search: names of compounds	No links
Databases of individual classes of compounds		
BIOPEP-UWM	Text search: name; reference; activity; sequence search (including exact match); search based on InChIKey	No links
CancerPPD	Text search including name, origin or other terms associated with peptide; Sequence search; Structure search: SMILES	No links
Carotenoids Database	Text search: compound name; Browse list of compounds	Links to KEGG
Phenol-Explorer	Text search: names of compounds and organisms; Browse lists of compounds and organisms	Links from FooDB Links to PubChem
USDA Flavonoids	Browse list of compounds	No links
USDA Isoflavones	Browse list of compounds	No links
USDA Proanthocyanidins	Browse list of compounds	No links
General databases of compounds with low molecular weight		
ChEMBL	Text search: names of compounds and targets, Browse list of targets Structure search: input as a SMILES or via molecular editor, exact match or similarity search Search based on amino acid sequences–exact match or similarity search	Links from ChemSpider, PubChem, DrugBank, FooDB Links to PubChem, ChemSpider, BRENDA ***, UniProt **, Open Targets **
ChemSpider	Text search: chemical name, common name or InChIKey Structure search using SMILES, InChI or molecule editor	Links to compound data in many databases (e.g., PubChem, ChEMBL, DrugBank, FooDB)
PubChem	Text search: chemical name, common name or InChIKey Structure search using SMILES, InChI or molecule editor Opportunities of search via exact match, similarity, substructure or superstructure	Links from many databases (e.g., ChemSpider, ChEMBL, KEGG, BRENDA, DrugBank, FooDB) Links to ChEMBL, ChemSpider
Databases of enzymes		
BRENDA	Text search: ligand name, enzyme name Structure search: molecule editor, option “substructure search”	Links from ChEMBL; Links to PubChem, UniProt **
OpenTargets	Text search including enzyme name and EC number	Links from ChEMBL **
Database of proteins		
UniProt	Text search: protein name, organism name Search based on amino acid sequence by similarity	Links from DrugBank **, DrugCentral **, Super Drug **, Cancer Resource **, canSAR **, NPACT **, NPCARE **, ChEMBL **, BRENDA ** Links to: ChEMBL **,
Metabases		
LabWorm	Browse lists of databases, classified according to content	Links to homepages of individual databases
MetaComBio	Browse lists of databases, classified according to content	Links to homepages of individual databases

* List of links restricted to databases summarized in this article. ** Links to target data. *** Links to compound and target data.

## Abbreviations

B16F10	Symbol of mouse skin melanoma cells
BLAST	Basic Local Alignment Search Tool
BRENDA	Braunschweig Enzyme Database
CAS	Chemical Abstracts Service
ChemRTP	Chemical Real Time Predictions
CID	Compound Identifier (in PubChem database)
EMBL	European Molecular Biology Laboratory
InChI	International Chemical Identifier
InChIKey	Key of International Chemical Identifier
IUPAC	International Union of Pure and Applied Chemistry
KEGG	Kyoto Encyclopedia of Genes and Genomes
MetaComBio	Meta Compound Bioactivity
NPACT	Naturally Occurring Plant-based Anti-cancer Compound-Activity-Target
NPCARE	Natural Products for CAncer gene REgulation
QSAR	Quantitative Structure-Activity Relationship
R	Absolute configuration of substituents around asymmetric carbon atom-*Rectus*
S	Absolute configuration of substituents around asymmetric carbon atom-*Sinister*
SMILES	Simplified Molecular Input Line Entry System or Simplified Molecular Input Line Entry Specification
USDA	United States Department of Agriculture
UWM	University of Warmia and Mazury
